# Gut–liver–hypothalamus axis dysfunction in mycotoxin toxicity: mechanisms and protective roles of natural compounds

**DOI:** 10.3389/fvets.2026.1852579

**Published:** 2026-06-03

**Authors:** Wael Ennab, Mohamed Tharwat, Abdallah A. Mousa, Hao-Yu Liu, Ghaid J. Al-Rabadi, Baiome Abdelmaguid Baiome, Fahad A. Alshanbari, Demin Cai

**Affiliations:** 1 Jiangsu Key Laboratory of Animal Genetic Breeding and Molecular Design, College of Animal Science and Technology, Yangzhou University, Yangzhou, China; 2 Department of Clinical Sciences, College of Veterinary Medicine, Qassim University, Buraidah, Saudi Arabia; 3 Animal Production Department, Faculty of Agriculture, Benha University, Banha, Egypt; 4 Laboratory of Gastrointestinal Microbiology, College of Animal Science and Technology, Nanjing Agricultural University, Nanjing, China; 5 International Joint Research Laboratory in Universities of Jiangsu Province of China for Domestic Animal Germplasm Resources and Genetic Improvement, Yangzhou, China; 6 Faculty of Agriculture, Mutah University, Karak, Jordan; 7 Parasitology and Animal Diseases Institute, National Research Centre, Giza, Egypt; 8 Department of Medical Biosciences, College of Veterinary Medicine, Qassim University, Buraidah, Saudi Arabia

**Keywords:** gut microbiota, gut–liver–hypothalamus axis, hepatotoxicity, mycotoxins, natural products, oxidative stress, probiotics

## Abstract

Mycotoxins, secondary toxic metabolites produced by filamentous fungi, pose significant health risks through contamination of food commodities such as grains. Major mycotoxins, including T-2 toxin, aflatoxins, and ochratoxin, are associated with a range of adverse health effects, particularly gastrointestinal disorders and hepatotoxicity. Increasing attention has been directed toward the gut–liver axis, which plays a critical role in mediating the systemic impact of these toxins, alongside growing interest in natural products as potential therapeutic agents. Natural compounds, including probiotics and antioxidants, have demonstrated the ability to mitigate mycotoxin toxicity through multiple mechanisms, such as toxin binding, inhibition of intestinal absorption, and biotransformation, mediated by pathways including Nrf2–ARE signaling and modulation of antioxidant enzyme activities. This review highlights the complex molecular mechanisms and signaling networks involved in mycotoxin toxicity, with particular emphasis on the gut–liver–hypothalamus axis. Disruption of gut microbiota homeostasis is recognized as a key initial event that compromises intestinal barrier integrity, facilitating toxin translocation to the liver and subsequent effects on hepatic function and hypothalamic hormonal regulation. Emerging evidence supports the potential of natural products as promising therapeutic strategies for restoring the balance of the gut–liver–hypothalamus axis and improving overall health outcomes; however, further research is needed to better elucidate their mechanisms of action and expand their application in the prevention and management of mycotoxin-related diseases.

## Graphical abstract

**Figure:**
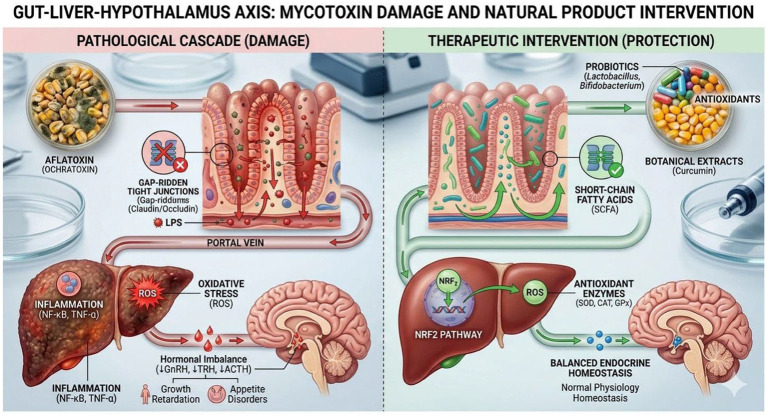

## 1 Introduction

Mycotoxins are secondary toxic metabolites naturally produced by certain filamentous fungi. More than 400 mycotoxins, such as T2, Aflatoxins, ochratoxin A, fumonisins, and deoxynivalenol, have been identified. These mycotoxins can contaminate various food commodities such as grains, nuts, and dried fruits, posing a serious health risk to humans and animals (1). These toxins are strictly linked to a range of adverse health effects, including gastro intestinal tract (GI-tract) disorders, liver damage, kidney dysfunction, and even cancer (2), Hence, strict monitoring and control measures are crucial in toxic contamination in food and feed products.

Manipulation of mycotoxins involves various strategies to reduce its levels. Some common methods including physical removal, chemical detoxification, biological control, and agricultural practices. These approaches aim to prevent mycotoxin exposure and protect public health (3). Additionally, ongoing research focused on developing innovative technologies and solutions to combat mycotoxin contamination effectively. Prior to 1985, the Food and Agriculture Organization estimated that global food crop contamination from mycotoxins was around 25% annually (4). Currently, the figure may be up to 60–80%, based on detectable levels, therefore, It is almost impossible to avoid toxins even at the lowest levels (1). Hence, the therapeutic Strategy of natural products against mycotoxins effects gained the scientist interest over the last 2 decades.

Natural products have shown promising potential in combating the effects of mycotoxins. The interest in exploring the use of natural products continues to grow as a way to mitigate the harmful impact of mycotoxins on human and animal health. This approach offers a potential solution to counteract the adverse health effects of mycotoxin exposure (5). Efforts in this area aim to enhance food safety and security by leveraging the beneficial properties of natural products to combat mycotoxin contamination effectively. Natural products including botanical extracts, essential oils, enzymes, probiotics, and antioxidants can neutralize mycotoxins and reduce their toxic effects. These natural products act through various mechanisms such as binding to mycotoxins, inhibiting their absorption, or promoting their degradation (6). By utilizing natural products, it is possible to limit the health risks posed by mycotoxin contamination in food and feed products. For example, some secreted biosurfactants like homologue of outer membrane protein A from bacteria Pantoea spp. can modulate binding/immobilization of aflatoxin B1 (7). Another research studied the effect of curcumin against AFB1 mutagenicity by Ames, *in vivo* micronucleus, comet and chemiluminescence, and plastic transformation methods (8). The results showed that curcumin was able to protect cells against mutagenicity. They also demonstrated a significant reduction in DNA damage through the stimulation of DNA repairs. Curcumin was reported by many authors to exhibit anticarcinogenic, antiproliferative and antimutagenic effects against various mutagens *in vitro* and *in vivo* (9, 10). Continued research and development in this field are essential to reveal new natural products that can offer protection against mycotoxins and contribute to a safer food supply chain. The potential of natural products in mitigating mycotoxin-related health issues is a promising area that warrants further exploration and investment.

The liver is a paramount organ in the human and animal body, orchestrating a myriad of essential functions that are fundamental to maintaining overall health. Among its many roles, liver detoxification emerges as a pivotal process, safeguarding the body against the harmful effects of xenobiotics, environmental toxins, and metabolic pathways (11). Beyond its immediate role in neutralizing exogenous toxins and as a central hub in the body’s metabolic network, the liver influences energy metabolism, nutrient utilization, and the regulation of blood. Moreover, it is intrinsically linked to the metabolism of endogenous (12, 13). Therefore, disruptions in these detoxification pathways can lead to the accumulation of harmful intermediates, potentially contributing to a range of health disorders, including liver diseases, hormonal imbalances, and increased susceptibility to chronic illnesses (14).

Generally, the liver detoxification process encompasses a cascade of enzymatic reactions, collectively known as phase I and phase II detoxification pathways. During phase I, the liver enzymes, such as the cytochrome P450 family, initiate the transformation of lipid-soluble toxins into intermediate metabolites (15). Subsequently, in phase II, these intermediates undergo conjugation with water-soluble molecules, facilitating their excretion from the body through bile or urine. This orchestrated interplay of enzymatic reactions ensures the efficient and comprehensive elimination of a diverse array of toxic compounds (16). The gut microbiome comprises a vast and diverse community of microorganisms, including bacteria, viruses, fungi, and archaea, the gut microbiome has emerged as a critical regulator of various aspects of both human and animal health (17). Among its multifaceted impacts, an increasingly recognized domain is its profound influence on liver function.

In the wake of a rapidly evolving understanding of the intricate interplay between environmental exposures and human health, recognizing the pivotal role of Gut-liver Axis becomes imperative. This article delves into the nuanced mechanisms underlying liver detoxification, shedding light on the intricate web of molecular pathways and signaling networks that contribute to this fundamental biological process. A comprehensive exploration of the significance of Gut microbiome and liver detoxification and recognizing the role of the Gut-Liver Axis in these pathologies, not only deepens our understanding of disease mechanisms but also opens avenues for therapeutic interventions aimed at restoring the balance within this complex interorgan communication system. Therefore, in this comprehensive review, we aim to underscore the critical importance of maintaining a healthy and functional liver for overall well-being.

## 2 Interspecies considerations in mycotoxin-induced gut-dysbiosis and liver detoxification

Before reviewing the effects of mycotoxins on gut microbiota and intestinal barrier function, it is critical to acknowledge that substantial anatomical, physiological, and metabolic differences exist across species. These differences profoundly influence mycotoxin absorption, distribution, metabolism, excretion, and toxicity, as well as microbiota composition and responses to dysbiosis (18). For example, the gastrointestinal tract of poultry (chickens, turkeys, ducks) differs markedly from that of monogastric mammals (pigs, mice, rats, rabbits) in terms of transit time, pH, and microbial composition (19). Expression and activity of cytochrome P450 (CYP) enzymes which are central to mycotoxin detoxification, vary more than 10-fold across species for specific mycotoxins (20). Furthermore, baseline gut microbiota composition, particularly the Firmicutes/Bacteroidetes ratio, ranges from approximately 10:1 in mice to 0.5:1 in chickens (21, 22). Therefore, results obtained in one species cannot be directly extrapolated to another species or to humans without independent validation (23).

The gut-liver axis varies substantially across species due to differences in portal anatomy, hepatic blood flow, bile acid composition and circulation, and expression of transporters and detoxification enzymes (24). Pigs are often considered the most translational model for human liver metabolism due to similarities in CYP450 expression, bile acid composition, and liver-to-body weight ratio (25). However, pigs are also highly sensitive to certain mycotoxins (e.g., DON and zearalenone) compared with rodents, which complicates direct extrapolation (26). Poultry (chickens, ducks, turkeys) have a unique hepatic portal system and significantly lower overall CYP450 activity, which may explain their differential susceptibility to some mycotoxins (e.g., relative resistance to AFB1 but sensitivity to ochratoxin A) (27–29).

Therefore, when interpreting gut-liver axis data across species, one must consider not only the effects of mycotoxins on intestinal permeability and dysbiosis but also the species-specific capacity of the liver to process those toxins and microbial products. The following sections present findings with explicit attention to these differences and readers are cautioned against direct cross-species generalization.

## 3 Mycotoxins lead to microbiota dysbiosis and intestinal barrier dysfunction

**Figure 1:**
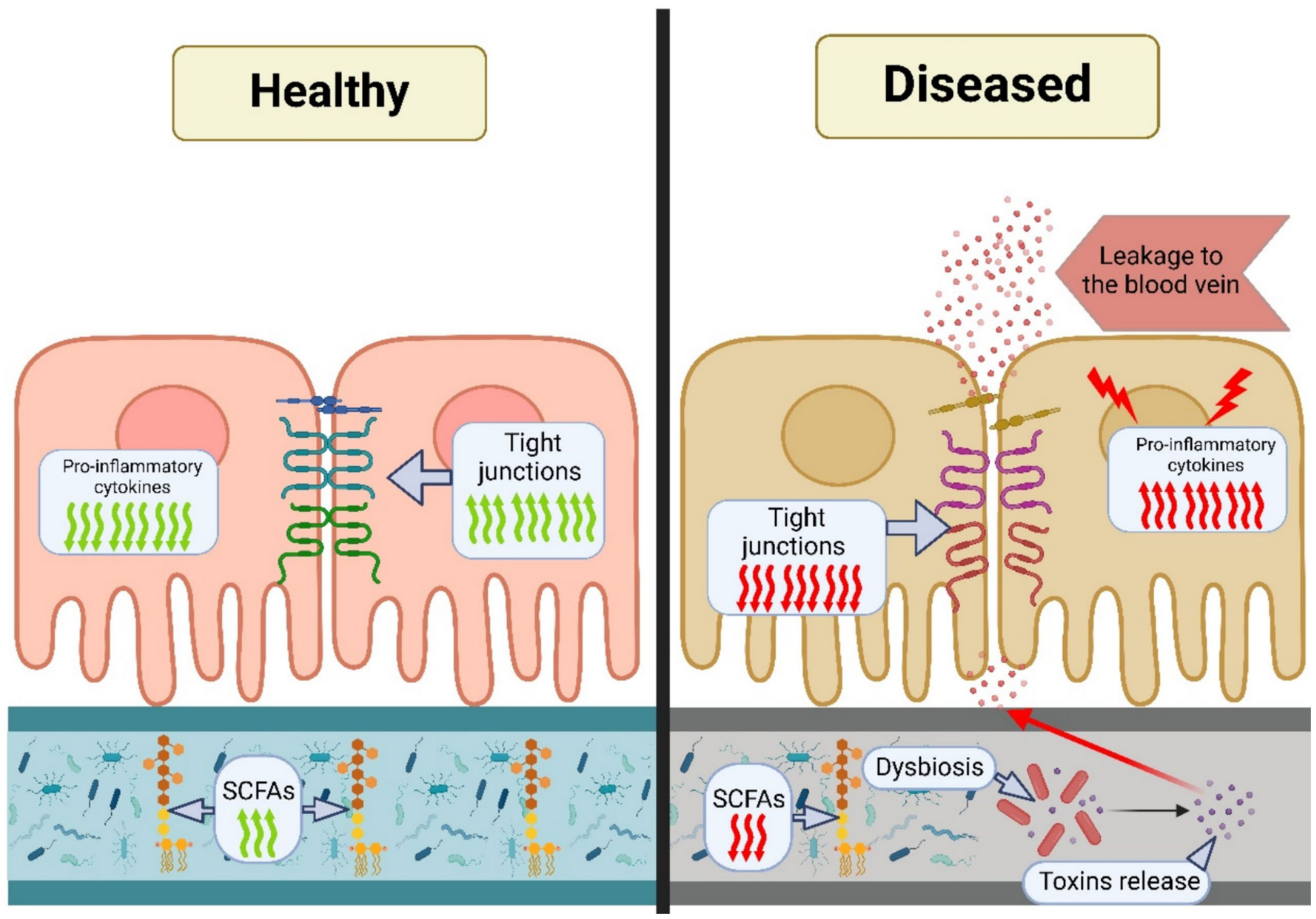
The effects of mycotoxin ingestion on the gut microbiota and intestinal barrier integrity. Mycotoxin exposure induces gut dysbiosis, leading to disruption and weakening of intestinal tight junctions, increased intestinal permeability (“gut leakage”), and enhanced production of pro-inflammatory cytokines. These alterations contribute to intestinal inflammation and impaired gut homeostasis.

The interaction between mycotoxins and the gut microbiome significantly impacts the integrity of intestinal barriers and microbiota composition. Mycotoxins, such as aflatoxins and fumonisins, have been shown to disrupt the tight junctions of the intestinal epithelium, leading to increased permeability and compromising the gut barrier function (30). This disruption can result in the translocation of toxins and harmful bacteria from the gut into the bloodstream, triggering inflammatory responses and systemic health issues (31, 32). Furthermore, mycotoxins can alter the composition and diversity of the gut microbiota, affecting the balance of beneficial and pathogenic microorganisms. These changes in the gut microbiome can have far-reaching consequences on overall health, as the microbiota plays a crucial role in various physiological processes, including digestion, immune function, and metabolism (33). Imbalances in the gut microbiota, known as dysbiosis, have been linked to compromised tight junction function, leading to increased intestinal permeability or “leaky gut.” Which is in order linked to a range of health conditions, such as inflammatory bowel disease, obesity, and metabolic disorders (34, 35). Recent research highlighted the critical role of the gut microbiota in maintaining the integrity of the intestinal barrier, primarily through its influence on tight junctions (TJs) (36). Tight junctions are crucial components of the intestinal epithelial barrier, acting as gatekeepers that regulate the permeability between the intestinal lumen and the bloodstream (37). They are composed of various proteins, including claudins, occludin, and junctional adhesion molecules, which work together to seal the spaces between epithelial cells. Moreover, Dysbiosis can trigger an inflammatory response, characterized by the release of pro-inflammatory cytokines such as TNF-*α*, IL-6, and IFN-*γ*, these cytokines can directly affect the expression and distribution of TJ proteins (38). For instance, TNF-α has been shown to downregulate the expression of occludin and claudins, leading to weakened TJs and increased permeability (39). In addition, the metabolites produced by gut bacteria, such as short-chain fatty acids (SCFAs), play a significant role in maintaining TJ integrity. SCFAs like butyrate have been demonstrated to enhance the expression of TJ proteins and promote barrier function (40). Conversely, a decrease in beneficial SCFA-producing bacteria can lead to diminished TJ integrity (41). Hence, the overgrowth of pathogenic bacteria due to dysbiosis can lead to the production of toxins and other harmful substances that disrupt TJ function (Figure 1). For example, enteropathogenic *Escherichia coli* produces proteins that can directly interfere with TJ assembly and function (42). Furthermore, Dysbiosis can increase oxidative stress within the intestinal environment, leading to increase the production of reactive oxygen species (ROS), which can damage TJ proteins and disrupt their function (43). (Table 1). Therefore, understanding the impact of mycotoxins on the gut microbiome and intestinal barriers is essential for developing strategies to mitigate their harmful effects and preserve gut health. By elucidating the mechanisms underlying these interactions, researchers can identify novel approaches to protect against mycotoxin-induced damage and promote a healthy gut environment.

**Table 1:** Mycotoxins-induced gut dysbiosis.

Animal species	Mycotoxin exposure	Analysis method	Analyzed sample	Effects	References
Pigs	DON: 1, 3 mg/kg feed for 28 days	16S rRNA gene sequencing	Small intestinal lumen digesta	In the duodenum and ileum, Firmicutes decreased while Actinobacteria increased. Among the genera, Lactobacillus and Cupriavidus were decreased, and Staphylococcus increased, while Burkholderia decreased in the duodenum and jejunum but increased in the ileum.”	(183)
Pigs	DON: 12 μg/kg, ZEN: 40 μg/kg, ZEN + DON: 40 + 12 μg/kg (BW) oral, for 42 days	EcoPlate tests	Ascending colon digesta	Lactic acid bacteria predominant flora. The number of mesophilic aerobic bacteria was decreased. The levels of *E. coli*, *C. perfringens*, and Enterobacteriaceae family were decreased.	(184)
Broiler	AFB1: 40 μg/kg feed for 21 days	Bacterial culturew	Ileal digesta	No effect on Bifidobacteria, Lactobacilli, *E. coli*, and *C. perfringens* were found	(185)
Broiler	AFB1: 1, 1.5, 2 mg/kg feed for 21 days	Bacterial culture	Cecal digesta	Increased total Gram-bacteria, aerobic bacteria, affect total lactic acid bacteria	(186)
Broiler	FB1 + FB2: 10.4 + 8.2 mg/kg, and FB3; 2 mg/kg, feed for 15 days	16S rRNA gene sequencing	Ileal digesta	Increased total *Clostridium perfringens*. And reduced the abundance of Lactobacillus spp., and Candidatus Savagella	(187)
Duck	OTA:235 μg/kg (BW) oral, for 14 days	16S rRNA gene sequencing	Excreta	Increased Bacteroidetes at the phylum level, Bacteroides at the genus level, and *Bacteroides plebeius* at the species level	(188)
Rabbit	ZEN: 400, 800, 1,600 μg/kg (BW) oral, for 28 days	16S rRNA gene sequencing	Cecal digesta	Reduced abundance of Actinobacteria, Lactobacillus, Blautia, Adlercreutzia, Oxalobacter, Desulfitobacter, s, and p-75-a5 And increased the abundance of Synergistetes, Proteobacteria, and Cyanobacteria	(189)
Mice	DON: 1.0 and 5.0 mg/kg, (BW) oral, every 2 days for 14 days	shotgun sequencing	Cecal digesta	Increased the Firmicutes phylum at low doses, and the most abundant genera were Mastadenovirus, Lactobacillus, Bacteroides, Parabacteroides, and Mucispirillum While Bacteroidetes phylum relative abundance were increased at high doses	(190)
Mice	ZEN: 10 mg/kg (BW) oral, for 14 days	16S rRNA gene sequencing	Colon digesta	The dominant bacteria at the phylum level in the colon were Firmicutes, Bacteroidetes, Proteobacteria, and Actinobacteria While reduced diversity of the microbiota Reduced abundance of Firmicutes, Bacteroidetes	(191)
Rat	DON: 100 μg/kg. (BW) oral, for 28 days	RT-PCR	Excreta	Increased the abundance of Prevotella and Bacteroides genera on day 10–20 On day 27, the expression of *Escherichia coli* was reduced	(192)
Rat	DON: 60, 120 μg/kg, (BW) oral, for 40 days	16S rRNA gene sequencing	Cecal digesta	At phylum level, Firmicutes and Bacteroidetes were the most abundant Increase in the relative abundance of Coprococcus genus	(193)
Turkey	OTA: 199 to 462 μg/kg feed, for 21, 42, 63, 105 days	Bacterial culture	Jejunum and cecal digesta, excreta	Reduced Lactobacillus and Bifidobacterium genera in the intestinal content and the excreta samples after 15 weeks	(194)

## 4 Gut-liver axis

The liver receives about 70% of its blood supply from the portal vein, which drains the GI-tract, pancreas, and spleen (44). This direct blood flow means that substances absorbed from the gut, including nutrients, toxins, and microbial products, are transported to the liver for processing. The gut microbiome plays a pivotal role in influencing liver function, highlighting the intricate interplay between the two organs through the Gut-Liver Axis (45). It starts with intestinal dysbiosis, which increases the intestine permeability by weakening tight junction barriers, and then the toxic substances reach the liver via bloodstream. This bidirectional communication system involves an array of molecular signals, including Pathogen-Associated Molecular Patterns, Damage-Associated Molecular Patterns, Immunoglobulin A, and Inflammatory Cytokines (46, 47). Microbial metabolites such as SCFAs and bile acids can modulate the expression and activity of hepatic detoxification enzymes, thereby impacting the efficiency of toxin processing (48). Moreover, immune modulators like T Regulatory Cells, Interleukin-10 (IL-10), Toll-Like Receptors, and macrophages play a crucial role in this complex interplay (49). The gut microbiome residing in the gastrointestinal tract exerts significant effects on liver physiology and function, with implications for various health conditions like non-alcoholic fatty liver disease (NAFLD), liver cirrhosis, and metabolic syndrome (50–52). The gut-liver axis represents a complex and bidirectional relationship between the gastrointestinal tract and the liver, mediated by portal circulation, immune signals, and microbial interactions. Understanding this intricate connection is critical in assessing how environmental exposures impact health, as the gut and liver collaborate to metabolize and detoxify various substances, including dietary components, drugs, and environmental toxins. Therefore, the intricate dynamics of the Gut-Liver Axis is essential in understanding the impact of environmental exposures on health.

### 4.1 The impact of dysbiosis on liver detoxification

The liver is a central organ in detoxifying harmful substances from the body. When toxic substances such as Lipopolysaccharides (LPS) produced by gut microbiota reach the liver through the bloodstream, they can significantly impair liver detoxification mechanisms (53). LPS, a component of Gram-negative bacterial cell walls, can enter the bloodstream due to increased intestinal permeability. In the liver, LPS binds to Toll-like receptor 4 (TLR4) on hepatocytes and Kupffer cells (54). This binding triggers an inflammatory response, activating nuclear factor-kappa B (NF-κB) and mitogen-activated protein kinases (MAPKs), leading to the production of pro-inflammatory cytokines such as TNF-*α*, IL-1β, and IL-6 (55). Chronic inflammation hampers the liver’s detoxification processes by causing hepatocellular injury and dysfunction (56). Moreover, dysbiosis increases the production of ROS within cells through various mechanisms, including mitochondrial dysfunction, activation of NADPH oxidases, and inhibition of antioxidant enzymes (57). ROS accumulation is associated with oxidative stress, causing damage to cellular components such as lipids, proteins, and DNA, contributing to tissue injury and dysfunction (3). ROS also can damage liver cells and mitochondria, impairing the cellular machinery responsible for detoxification, including enzymes involved in Phase I and Phase II detoxification pathways (58). The liver Phase I detoxification involves CYP enzymes that oxidize toxins, making them more water-soluble. Inflammation and oxidative stress can downregulate the expression and activity of CYP enzymes, reducing the liver ability to metabolize and clear toxins (59). The liver Phase II detoxification involves conjugation reactions, where toxins are linked to substances like glutathione to facilitate their excretion (60). Dysbiosis-related inflammation can deplete glutathione levels, limiting the liver capacity to conjugate and eliminate toxins.

The mechanisms underlying apoptosis involve multiple pathways, including the mitochondrial pathway, death receptor pathway, and endoplasmic reticulum stress pathway. These pathways converge on the activation of caspases, which execute the apoptotic program by cleaving cellular substrates and dismantling the cell (61). Apoptosis induction by mycotoxins contributes to tissue damage, organ dysfunction, and the pathogenesis of mycotoxin-related diseases (4). Furthermore, the endoplasmic Reticulum (ER) Stress by unfolded Protein Response (UPR) crucial role in protein folding and detoxification processes (62). Toxins from gut microbiota can induce ER stress, triggering the unfolded protein response. Chronic ER stress impairs the liver’s ability to process and detoxify xenobiotics and endogenous toxins, leading to cellular dysfunction and apoptosis (63). Additionally, Toxins and ROS can damage mitochondrial DNA and membranes, leading to impaired ATP production. ATP depletion compromises the liver’s metabolic functions, including the energy-dependent processes of detoxification (64). The microbiota toxic substances lead to macrophage activation in the Kupffer cells, the liver’s resident macrophages, become activated by LPS and other microbial products, producing ROS and pro-inflammatory cytokines. Elevating the levels of cytokines such as TNF-*α*, IL-1β, and IL-6 due to gut-derived toxins may contribute to chronic inflammation (65, 66). These cytokines interfere with the expression of detoxification enzymes and transporters, reducing the liver’s detoxification capacity and promoting fibrosis and cirrhosis. Moreover, gut dysbiosis can alter bile acid metabolism, leading to the accumulation of toxic bile acids in the liver (67). These bile acids can damage hepatocytes and cholangiocytes (bile duct cells), disrupt bile flow and impair the excretion of conjugated toxins through bile.

### 4.2 The mechanisms and signaling pathways undergoing liver detoxification

When toxins molecules reach to liver through the vein. These toxins lead to disruption in the roles of cytochrome P450 (CYP450) in xenobiotic detoxification and activation, changes in CYP450 expression and related genes can significantly affect xenobiotic potential risk (10). Moreover, toxins substances can modulate immune responses by influencing the function of immune cells and cytokine production resulting in activating inflammatory Signaling Pathways.

The mitogen-activated protein kinase (MAPK) family comprises three major subfamilies with distinct and sometimes opposing functions: extracellular signal-regulated kinase 1/2 (ERK1/2), c-Jun N-terminal kinase (JNK), and p38 MAPK. A common oversimplification is to label all MAPKs as uniformly “pro-inflammatory” or “pro-apoptotic (68).” In reality, their effects are highly context-dependent, varying with cell type, stimulus intensity, duration, and cross-talk with other pathways. While, ERK1/2 Typically activated by growth factors and mitogens, ERK signaling is generally pro-survival and anti-apoptotic. In the liver, ERK promotes hepatocyte proliferation and regeneration following injury (69). However, sustained ERK activation can contribute to fibrosis and hepatocellular carcinoma. The JNK known as the most context-sensitive MAPK. Acute JNK activation can be pro-apoptotic (e.g., in response to TNF-*α* or oxidative stress), but it also plays essential roles in cell survival, metabolism, and tissue repair depending on the duration of activation and the cellular environment (70). In mycotoxin-induced liver injury, JNK has been reported to both promote and limit damage. Notably, the p38 MAPK known Similarly to have dual-function. p38 is classically associated with pro-inflammatory cytokine production (e.g., IL-1β, TNF-*α*) and stress responses (68). However, p38 also regulates anti-inflammatory pathways, cell cycle arrest, and differentiation. In hepatocytes, p38 can either exacerbate or protect against toxin-induced cell death depending on the specific isoform (p38α, p38β, p38γ, p38δ) and the timing of activation.

Therefore, cytokine production resulting in activating inflammatory signaling pathways as mentioned above (71, 72). The activation of these pathways regulates various cellular processes such as proliferation, differentiation, apoptosis, and inflammation. Mycotoxin-induced MAPK and NF-κB signaling contributes to the modulation of cellular responses to stress and the regulation of gene expression associated with toxicity and cellular adaptation (73). Activation of these pathways promotes the key of pro-inflammatory cytokines (IL-1, IL-6, and TNF-*α*) and chemokines (CXC, CC, CX3C, and XC), exacerbating inflammations and tissue injury. Nuclear factor-κB (NF-κB) and Nrf2 pathways subsequently to detoxify and eliminate exogenous chemicals and their metabolites. Hence, increase the levels of AST, ALT, ALP, LDH, and *γ*-GT which is a sign of liver dysfunction (74).

The Nrf2 (nuclear factor erythroid 2-related factor 2)–Keap1 (Kelch-like ECH-associated protein 1)–ARE (antioxidant response element) signaling pathway is a master regulator of cellular defense against oxidative and electrophilic stress. Under basal conditions, Nrf2 is sequestered in the cytoplasm by Keap1, which facilitates its ubiquitination and proteasomal degradation, maintaining low basal activity. Upon exposure to mycotoxins or other stressors, Keap1 cysteine residues are modified, allowing Nrf2 to stabilize, translocate to the nucleus, and bind AREs. This drives transcription of antioxidant enzymes (e.g., SOD, catalase, GPx) and phase II detoxification enzymes (e.g., GSTs, UGTs, SULTs), thereby neutralizing ROS and enhancing toxin excretion (75–77).

However, the Nrf2 pathway is not exclusively beneficial. While transient, moderate activation is protective, chronic or excessive activation carries significant risks. The constitutive activation has been documented in various cancers, including hepatocellular carcinoma, where it promotes chemoresistance, metabolic reprogramming favoring tumor growth, and evasion of apoptosis (78, 79). Oncogenic mutations in *KEAP1* or *NRF2* itself have been identified in human liver cancers and are associated with poor prognosis. Therefore, therapeutic strategies targeting Nrf2 must aim for moderate, controlled activation to achieve hepatoprotection without tipping into pathological sustained activation. This duality is particularly relevant for natural products, many of which are mild, reversible activators.

**Figure 2:**
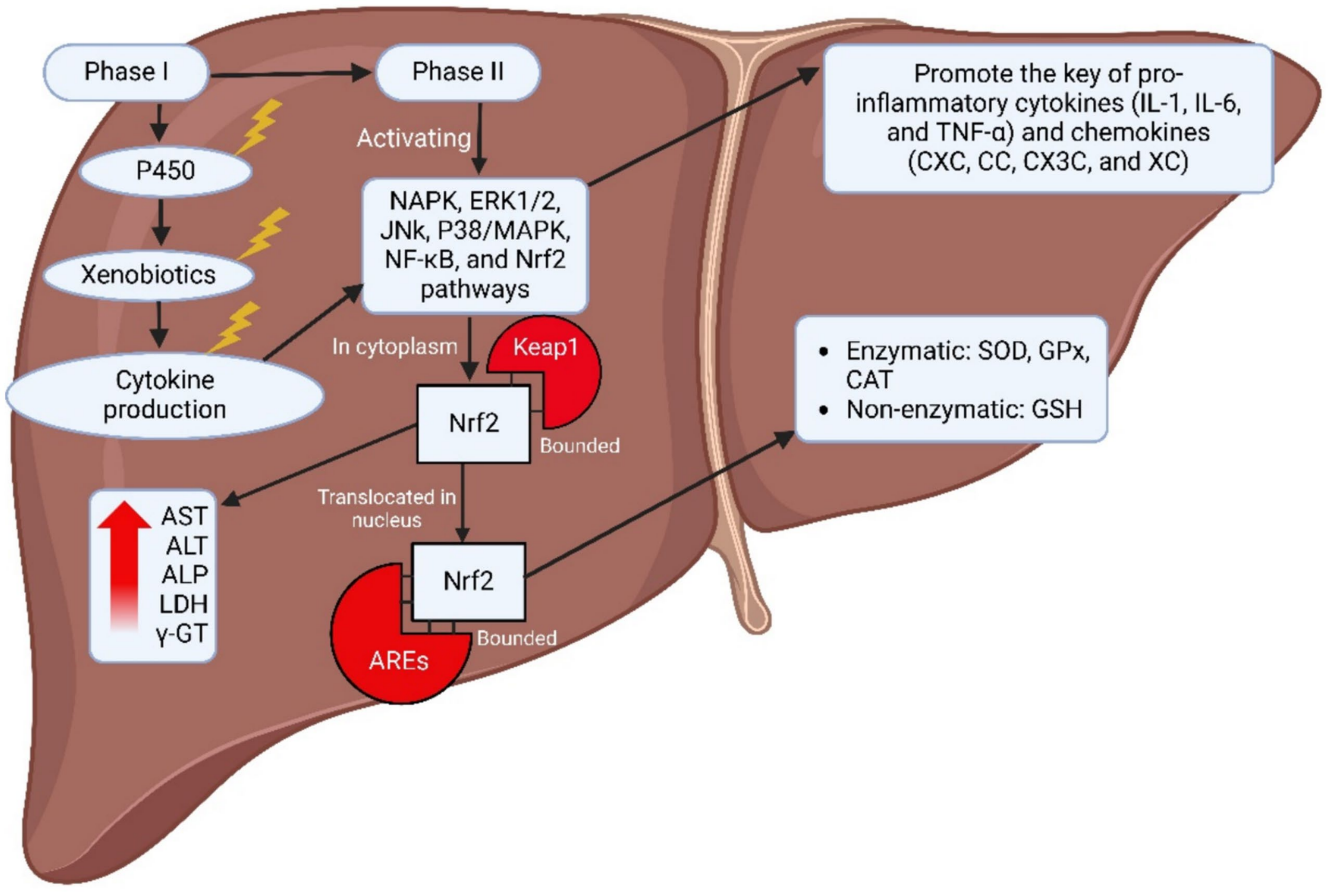
The mechanisms and signaling pathways undergoing liver detoxification.

By upregulating antioxidant defenses and phase II detoxification enzymes, Nrf2 helps mitigate the oxidative and electrophilic stress induced by these toxins, protecting the liver and maintaining its functional integrity (Figure 2) (Table 2). Understanding and potentially targeting this pathway could provide therapeutic strategies to enhance liver detoxification and protect against liver diseases associated with microbial dysbiosis.

**Table 2:** Gut microbiota-associated liver diseases.

Species	Mycotoxin exposure	Analysis method	Analyzed sample	Effects	References
Duck	OTA: 235 μg/kg, (BW) oral, for 14 days	Histopathology, biochemical analysis, WB, and 16S rRNA gene sequencing	Excreta and liver	OTA alters cecum microbiota diversity and composition, leading to an increase in Gram-negative bacterial-derived LPS and entrance into the blood and liver through defective intestinal barrier, and ultimately facilitates the development of liver inflammation in ducks	(188)
Mice	AFB1: 25 μg/kg, (BW) oral, for 28 days	Histological Analysis, biochemical analysis, WB, RT-qPCR, and 16S rRNA gene sequencing	Excreta, colon, and liver	The treatment of AFB1 in mice altered gut microbiota composition, such as increasing the relative abundance of Bacteroides, Parabacteroides, and Lactobacillus, inducing colonic barrier dysfunction and promoting liver pyroptosis	(195)
Broiler and pigs	DON: 5 mg/kg and 10 mg/kg, feed, for 14 days	Histological Analysis, blood biochemistry, and 16S rRNA gene sequencing	Jejunum digesta, liver, and kidney	Romboutsia and Bacteroides were dominant genera in broilers, while in pigs the relative abundance of Streptococcus and Prevotella_9 were decreased, leading to injury and damaged liver and kidney	(196)
Mice	OTA: 250 μg/kg, (BW) oral, for 21 days	Histological Analysis, biochemical analysis, ATP levels, WB, RT-qPCR, and 16S rRNA gene sequencing	Colonic digesta, liver, and colon	At the phylum level, OTA significantly reduced the relative abundance of Bacteroidetes while significantly increasing Firmicutes. While reduced Lactobacillus and Desulfovibrio relative abundance and increased Bacteroides at the genus level Insufficient production of intestinal SCFAs indicated that OTA caused intestinal dysbiosis resulting in increased Gram-negative bacterial-derived LPS that could enter into the blood and liver via defects on the intestinal barrier	(197)
Mice	T2: 1.6 mg/kg, (BW) oral, for 3 weeks	Histological Analysis, biochemical analysis, RT-qPCR, and 16S rRNA gene sequencing	Jejunum and liver	Gut dysbiosis marked by a decline in beneficial phyla, genera, and species. And an increase in pathogenic bacteria Induced oxidative stress, inflammation, and disrupted histology. Impaired lipid metabolism by downregulating PPAR pathway genes	(198)
Broiler	AFB1: 100 μg/kg, feed, for 7 days	Histological Analysis, biochemical analysis, RT-qPCR, LC–MS/MS Analyses, and 16S rRNA gene sequencing	Duodenal content, liver and duodenum	Significant decrease in the abundance of Firmicutes and Proteobacteria and a significant increase in the abundance of Tenericutes and Verrucomicrobia AFB1 mainly disrupted the abundance of bacteria associated with bile acid metabolism, such as Faecalibacterium, Subdoligranulum Disruption in the tight junctions and damage in the duodenum Liver tissue damage, hepatocyte lipid droplet aggregation, and even steatosis, might promote abnormal lipid metabolism in the liver	(199)

## 5 Gut-liver-hypothalamus axis

**Figure 3:**
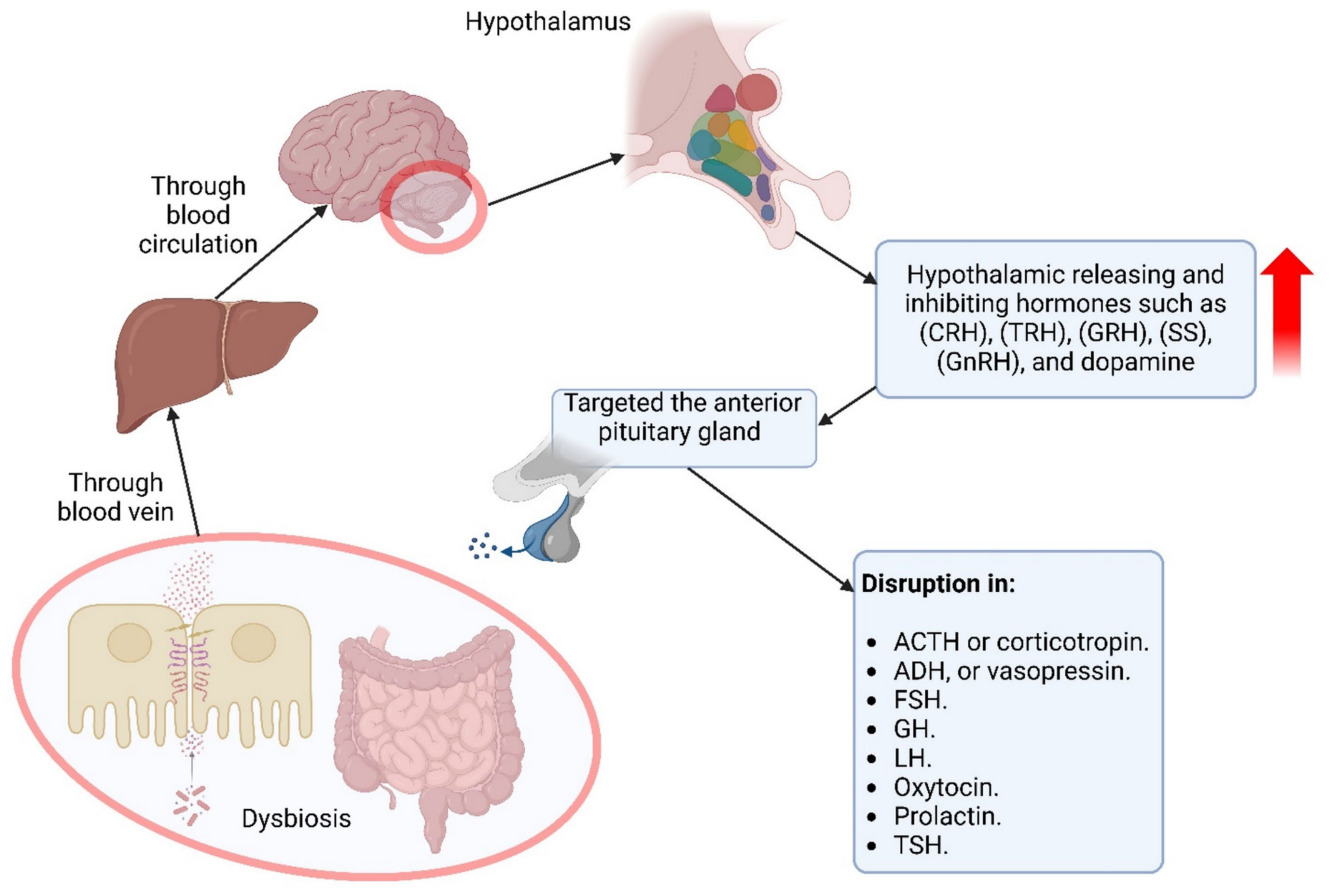
Dysbiosis affects hypothalamus released hormones, leading to disruption of the anterior pituitary gland and its hormones.

As mentioned previously, the Liver receives around 70% of its blood supply from the portal vein, which drains the GI-tract, pancreas, and spleen. Gut dysbiosis associated with an increase toxins or substances permeability of LPS and therefore affects the Liver functions, which means these substances will remain in the blood, hence, reaching every single organ in the body including the hypothalamus. Hypothalamus is known as the control panel of large numbers of hormones, hypothalamic releasing and inhibiting hormones such as corticotropin-releasing hormone (CRH), thyrotropin-releasing hormone (TRH), growth hormone-releasing hormone, somatostatin (SS), also called growth hormone-inhibiting substance, gonadotropin-releasing hormone (GnRH), dopamine, etc. (80, 81). These hormones target the anterior pituitary gland, to make a disruption in its related hormones such as adrenocorticotropic hormone, antidiuretic hormone, follicle-stimulating hormone, growth hormone, luteinizing hormone, oxytocin, prolactin, and thyroid-stimulating hormone (82, 83). It affects a vast number of biological responses such as physiological, psychological, and immunological, resulting in playing an important role in health and disease in animals and humans. Previous study, suggests that DON oral dose induced anorexic POMC via NF-κB activation by increasing the levels of proinflammatory cytokines in the hypothalamus and anorexic CCK production via increasing intestinal TRPA1 expression (84). Another study considered the negative impact of ZEA on the reproductive system by the involvement of the hypothalamic–pituitary-gonadal axis-associated reproductive function and the mRNA and protein expressions of hypothalamic GnRH were elevated by ZEA (85, 86). A dose-related decrease in the expression of all the hypothalamic neuropeptides studied was found in response to AFB1, leading to alteration inducing appetite disorders as a macroscopic effect (87). Moreover, prepubertal exposure to T-2 toxin advances pubertal onset and development in female rats via promoting the onset of hypothalamic–pituitary-gonadal axis function (88) (Figure 3) (Table 3). Therefore, the liver plays a pivotal role in the “Gut-Liver-Hypothalamus Axis” as it takes the central position in health and disease.

**Table 3:** Gut dysbiosis affect hypothalamus (gut-liver-hypothalamus axis).

Animal species	Mycotoxin exposure	Analysis method	Analyzed sample	Effects	References
Rat	AFB1: 150 μg/kg and 300 μg/kg, (BW) oral, for 5 weeks	Immunohistochemistry and Rt-qPCR	Brain sections at the hypothalamic level	Rats lost weight The expression of genes for multiple hypothalamic neuropeptides regulating food intake was significantly reduced	(200)
Zebrafish	15–75 ng/mL 7 dpf (exposed to water with a certain concentration of AFB1)	Whole mount immunostaining, Rt-qPCR, larval locomotion test, and viability and morphology after AFB1 exposure	zebrafish embryos	Reduced motor capacity and a significantly reduced startle response after the percussive stimulus PCR results showed that AFB1 decreased the gene expression of NGFA and PRTGA, and increased the gene expression of ATP1B1B	(201)
Silver catfish	AFB1: 1177 ppb kg feed−1, for 7, 14, and 21 days	Behavioral tests, BBB permeability to Evans Blue, AChE and Na+, K + -ATPase activities in brain synaptosome, histopathology, and protein content	Brain and hypothalamus	Decreased expression of neuropeptides in the anorexic hypothalamus, led to an imbalance between anorexigenic and orexigenic signals The neurogenic changes associated with appetite, resulted in disruption in neurotransmitter homeostasis, thereby affecting the brain synaptic transmission function	(202)
Mice	ampicillin: 10, 50, and 100 mg/kg, (BW) oral for 10 days	Immunoblotting, ELISA, Rt-qPCR, Immunofluorescence assay, Flow cytometry, LAL assay, and Pyrosequencing	Colon and hippocampal	The exposure of ampicillin caused monocyte/macrophage-activated colitis by increasing the population of Proteobacteria including *K. oxytoca*, resulting in anxiety	(203)
Mice	MSG: 3 mg/g (BW) oral, from day 2 to 8 once daily	Biochemical Analysis, Histology and Immunohistochemistry, Rt-qPCR, Metabolic Activity, and 16S rDNA Sequencing,	Feces, Hypothalamus and liver	Gut microbiota dysbiosis affected retinol metabolism pathway in the liver and neuronal damage in the brain, which jointly resulted in the development of abdominal obesity	(204)
Mice	High-sugar and butter (HSB) feed, for 12 weeks	Biochemistry, Histological evaluation, Rt-qPCR, and fecal microbiome analysis, and Marble-burying test	Feces, liver, and hypothalamus	Microbiome composition was associated with inter-correlated changes in animal compulsion-like and anxiety-like behaviors, and the regulation of genes related to food intake	(205)

## 6 Potential therapeutic strategies

The gut-liver-hypothalamus axis, a bidirectional communication pathway between the gut and liver linked with the hypothalamus. Therefore, modulating the gut microbiome using natural products offers a promising strategy to enhance liver detoxification. Such as probiotics, prebiotics, synbiotics, phytobiotics, and herbal Supplements (Table 4).

**Table 4:** Studies on natural products showed a beneficial effect on gut microbiota, liver, and hypothalamus.

Natural products	Effects	References
*Bifidobacterium infantis* 35,624	Reduces depressive-like behavior via alleviating 5-HT	(206)
*Lactobacillus farciminis*	Prevents gut barrier leakiness and reverses psychological stress-induced HPA axis activation	(207)
*Lactobacillus helveticus* and *Bifidobacterium longum*	Normalizes hippocampal BDNF levels and inflammation	(208, 209)
B-immuno galacto-oligosaccharide and Fructo-oligosaccharides	Stimulate the growth of beneficial bacteria Increased BDNF, and reduction of stress, pro-inflammatory cytokines, and corticosterone levels	(210, 211)
Resveratrol (Polyphenol)	Elevates nor epinephrine and 5-HT levels in pre frontal cortex and upregulates BDNF levels	(212, 213)
Blueberry (Anthocyanins)	Improved depression-like behavior and working memory and significantly increases brain activity	(214)
Korea red ginseng and urushiol from *Rhus verniciflua*	Attenuating ALD by downregulating TLR4 expression	(215)
Flaxseed oil	Modulating gut dysbiosis and reducing inflammatory cytokines	(216)
Rhubarb extract	Protecting the liver from oxidative stress and inflammation due to the modulation of the gut microbiota	(217)
Oats	Protection against alcohol-induced leaky gut by enhancing the integrity of colonic mucosa and tight junctions	(218)
Fish oil	Decreased the abundance of Bacteroidetes, Rikenellaceae, Bacillaceae, and Alistipes, Suppressed TLR4 activation and inhibited endotoxin production, and in chronic ethanol-fed rats	(219)
Garlic polysaccharide	Alleviate various biochemical indicators Increasing the abundance of Lactobacillus, Lachnospiraceae and decreasing the abundance of Firmicutes and Facklamia	(220)
Inulin	Increases SCFA-producing bacteria including Phascolarctobacterium, Lachnospiraceae, *Akkermansia muciniphila*, and Bacteroides	(221, 222)
Lycopene	Increase SCFAs producing bacteria such as Allobaculum and reduce destructive bacteria, including Firmicutes, Desulfovibrio, Lachnospiraceae_NK4A136_group, and Alistipes Lycopene might prevent NAFLD by attenuating gut microbiota dysbiosis and NF-κB/NLRP3 signaling pathway	(223)
Lycopene	Lycopene Attenuates T2 Mycotoxin-Induced Hepatotoxicity and Dysbiosis by Activating PPAR Signaling	(198)
Lutein	Decreased liver total triglycerides, cholesterol and ALT Increased HDL, Hepatic insulin sensitivity, and Hepatic fatty acids catabolism	(224)

### 6.1 Probiotics

Probiotics, which are live microorganisms that provide health benefits when administered in appropriate amounts, have emerged as a significant strategy for modulating the gut microbiome to improve intestinal health and enhance liver detoxification. The molecular mechanisms and signaling pathways underlying these benefits are complex and multifaceted, encompassing direct interactions with gut microbiota, strengthening of gut barrier function, modulation of immune responses, and attenuation of systemic inflammation. One of the primary mechanisms by which probiotics exert their beneficial effects is through the restoration of microbial balance in the gut in case of dysbiosis. Probiotics, such as Lactobacillus and Bifidobacterium species, can promote the growth of beneficial bacteria while inhibiting pathogenic organisms (89).

A specific probiotic strain has demonstrated distinct capacities for mycotoxin binding or degradation. For example, *L. rhamnosus* RC007 and *Bifidobacterium longum* LC67 bind aflatoxin B1 (AFB1) *in vitro*, but binding efficiency varies more than 10-fold among strains tested under identical conditions (90, 91). *Saccharomyces cerevisiae* RC016 reduces AFB1 bioavailability in rodent models, while other yeast strains show no detectable effect (92). Some *Lactobacillus* strains degrade deoxynivalenol (DON) via glycosylation, but this enzymatic activity is absent in closely related strains, underscoring the necessity of strain-level characterization (93). Critically, in vitro binding or degradation does not guarantee efficacy *in vivo* due to the complex gastrointestinal environment, including variable pH, proteolytic enzymes, bile salts, and competition from resident microbiota (30, 94).

In contrast, Probiotic effects are dose-dependent, with typical effective doses ranging from 10^8^ to 10^11^ colony-forming units per day in animal studies (95). However, higher doses are not universally better; excessive doses may cause dysbiosis, immune overstimulation, or even bacteremia in immunocompromised hosts (94, 96). A U-shaped or inverted-U dose–response curve is common, particularly for immune modulation, where both insufficient and excessive doses fail to produce protective effects (97). The live probiotics are generally more effective than heat-killed preparations for mechanisms requiring metabolic activity (e.g., SCFA production, immune modulation), although toxin binding may persist after heat inactivation (98). Encapsulation (e.g., alginate or chitosan microspheres) improves survival through gastric passage and enhances delivery to the lower intestine (99). Prophylactic administration (before mycotoxin exposure) is generally more effective than therapeutic administration (after exposure), particularly for binding-based mechanisms that prevent initial toxin absorption (100).

However, the interaction between probiotics and gut microbiota involves complex signaling pathways, including the modulation of microbial gene expression and metabolic activity. For instance, probiotics can influence the production of SCFAs by gut bacteria, which are key signaling molecules involved in maintaining gut and liver health (101). These SCFAs serve as signaling molecules that activate various pathways, including the G-protein-coupled receptors (GPCRs), GPR41, and GPR43, which mediate anti-inflammatory and metabolic effects (102). SCFAs also influence the activity of histone deacetylases (HDACs), thereby regulating gene expression and maintaining gut barrier integrity (103). Moreover, SCFAs can enter the bloodstream and exert systemic effects, including the modulation of liver function and enhancement of liver detoxification pathways (104). By supporting these processes, probiotics contribute to improved liver health and detoxification. Probiotics also play a critical role in enhancing the integrity of the gut barrier. It can increase the expression and function of tight junction proteins, such as occludin, claudins, and zonula occludens-1 (ZO-1), which are vital components of the gut barrier (105). The signaling pathways involved in this process include the activation of AMPK and the modulation NF-κB pathway (106). By reinforcing these tight junctions, probiotics help prevent the leakage of harmful substances. Additionally, probiotics stimulate the production of mucins, the glycoproteins that form a protective mucus layer over the intestinal epithelium (107), further strengthening the gut barrier and providing an additional line of defense against pathogens and toxins.

Immune modulation is another significant mechanism through which probiotics exert their effects. Probiotics can influence the immune system by promoting the production of anti-inflammatory cytokines, such as IL-10 and transforming growth factor-beta (TGF-*β*), while simultaneously reducing pro-inflammatory cytokines like TNF-*α* and IL-6 (108). This balanced immune response helps maintain immune homeostasis and reduces systemic inflammation, which is beneficial for both intestinal and liver health. Probiotics also affect the activity of various immune cells, including dendritic cells, macrophages, and T cells, enhancing the gut’s ability to respond appropriately to pathogens (109). The signaling pathways involved in this immune modulation include the Toll-like receptor (TLR) signaling pathway, which recognizes microbial-associated molecular patterns (MAMPs) and activates downstream signaling cascades that regulate immune responses (110). Reducing systemic inflammation is another key benefit of probiotics. By maintaining gut barrier integrity and reducing the translocation of LPS, LPS can trigger significant inflammatory responses primarily through the activation of the TLR4 signaling pathway, which leads to the production of pro-inflammatory cytokines (108). Probiotics can attenuate this pathway by competing with pathogenic bacteria for binding sites and modulating the gut microbiota composition. Probiotics also enhance the body’s antioxidant defenses by activating the Nrf2 pathway, which upregulates the expression of antioxidant genes and reduces oxidative stress (111).

Numerous studies have provided evidence supporting these mechanisms. Clinical trials on patients with NAFLD have shown that probiotic supplementation can improve liver enzymes, reduce liver fat accumulation, and decrease systemic inflammation (112, 113). For example, a randomized controlled trial demonstrated that probiotics containing Lactobacillus and Bifidobacterium significantly improved liver function tests and reduced inflammatory markers in NAFLD patients (114). Research has also highlighted the role of probiotics in enhancing gut barrier function. A study involving patients with irritable bowel syndrome (IBS) found that probiotics improved intestinal permeability and reduced symptoms, indicating their potential to strengthen the gut barrier and prevent the translocation of harmful substances (115, 116). Additionally, probiotics have been shown to modulate immune responses favorably. A study on children with atopic dermatitis found that probiotics reduced the severity of the condition by modulating the immune system and reducing inflammatory responses (117, 118). Animal studies have demonstrated that probiotics can increase the production of SCFAs, leading to beneficial effects on gut health and liver function. Mice treated with probiotics exhibited higher SCFA levels, improved gut barrier integrity, and enhanced liver detoxification pathways (119).

### 6.2 Prebiotics

Prebiotics are defined as substrates that are selectively utilized by host microorganisms conferring a health benefit (120). Prebiotics, such as inulin, fructooligosaccharides, and galactooligosaccharides, are dietary fibers that resist digestion in the upper gastrointestinal tract and selectively stimulate the growth or activity of beneficial bacteria in the colon (121, 122). These compounds play a pivotal role in modulating the gut microbiome, which in turn influences intestinal health and liver detoxification through complex molecular mechanisms and signaling pathways (121, 123).

However, “selective” does not mean universal. Different prebiotics enrich different bacterial taxa, and baseline microbiota composition determines individual responses (124). Moreover, effective prebiotic doses in animal studies typically range from 1 to 10% of dietary intake (125). Higher doses cause osmotic diarrhea, bloating, and abdominal discomfort in a dose-dependent manner (126). Chronic administration (generally >4–6 weeks) is required to observe stable microbiota shifts, although SCFAs increases may occur within days (127). Furthermore, prebiotics themselves are not absorbed in the small intestine; their effects are mediated entirely by microbial fermentation products, primarily SCFAs such as acetate, propionate, and butyrate (128). This makes prebiotics less susceptible to first-pass metabolism issues but highly dependent on individual gut microbiota composition, which varies considerably across individuals and species (129).

Beneficial gut bacteria, such as Bifidobacteria and Lactobacilli, ferment prebiotics to produce SCFAs like acetate, propionate, and butyrate. Butyrate, in particular, plays a crucial role in maintaining intestinal barrier integrity by enhancing mucin production and tightening tight junction proteins like occludin and claudins (130–132). SCFAs also act as signaling molecules by activating G-protein coupled receptors (GPCRs) such as GPR41 (FFAR3) and GPR43 (FFAR2) on gut epithelial cells, immune cells, and enteroendocrine cells (133, 134). Activation of these receptors modulates immune responses and inflammatory pathways. Furthermore, butyrate derived from prebiotic fermentation activates Nrf2 pathway in liver cells (135, 136). Nrf2 is a transcription factor that upregulates antioxidant enzymes (e.g., glutathione S-transferases, GSTs) and phase II detoxification enzymes (e.g., UDP-glucuronosyltransferases, UGTs) (137). These enzymes play crucial roles in neutralizing reactive oxygen species (ROS) and detoxifying xenobiotics and metabolic byproducts, thereby enhancing the liver’s detoxification capacity and reducing oxidative stress. Prebiotics also influence bile acid metabolism by modifying primary bile acids synthesized in the liver into secondary bile acids through interactions with gut bacteria (138). Bile acids serve as signaling molecules that activate nuclear receptors like the farnesoid X receptor (FXR) and Takeda G protein-coupled receptor 5 (TGR5) in the liver and intestine (139, 140). Activation of these receptors regulates lipid and glucose metabolism, promotes bile acid synthesis and transport, and influences energy metabolism and anti-inflammatory responses, thereby contributing liver health and metabolic homeostasis.

Additionally, prebiotics promote immune modulation and enhance gut barrier integrity by shaping the gut microbiota composition towards a more beneficial profile (141). This microbial shift increases the production of antimicrobial peptides and competes against pathogenic bacteria, strengthening the gut barrier and reducing epithelial permeability to toxins. Clinical studies and experimental models consistently support the beneficial effects of prebiotics on gut microbiome modulation, intestinal health, and liver function (142). Interventions with prebiotics have demonstrated improvements in markers of liver inflammation and reductions in liver fat accumulation in patients with conditions like NAFLD (143). Animal studies further highlight that prebiotic supplementation enhances detoxification enzyme activities and reduces oxidative stress markers in the liver, underscoring their therapeutic potential in liver health.

### 6.3 Phytobiotics and herbal supplements

Phytobiotics, including polyphenols (e.g., curcumin, resveratrol, quercetin), carotenoids (e.g., lycopene, lutein, β-carotene), and other plant-derived compounds—have demonstrated promising *in vitro* antioxidant and Nrf2-activating properties (144). These compounds act as prebiotics by serving as substrates for fermentation by gut microbiota (145, 146). This fermentation produces SCFAs such as acetate, propionate, and butyrate. SCFAs play crucial roles in maintaining gut barrier integrity by regulating tight junction proteins like occludins and claudins (103). Furthermore, phytobiotics enhance the activity of phase I and phase II liver detoxification enzymes (147). Phase I enzymes, including cytochrome P450 family members, initiate the oxidation of xenobiotics, while phase II enzymes like glutathione S-transferases and UDP-glucuronosyltransferases conjugate these metabolites for excretion (148). Phytobiotics also possess potent antioxidant properties. Polyphenols, for example, act as direct scavengers of free radicals and reactive oxygen species (ROS), thereby mitigating oxidative stress in liver cells (149). They also stimulate the production of endogenous antioxidants such as glutathione, which further protects hepatocytes from oxidative damage.

At the molecular level, phytobiotics activate several keys signaling pathways crucial for their beneficial effects on intestinal health and liver function. For example Nrf2 activation by phytochemicals triggers the expression of antioxidant response element (ARE)-mediated genes, including phase II detoxification enzymes and antioxidant genes (150). Additionally, phytobiotics activate the AMP-Activated Protein Kinase (AMPK) pathway (151). AMPK regulates cellular energy homeostasis and metabolism, supporting mitochondrial function and lipid metabolism in hepatocytes (152). Furthermore, phytobiotics modulate TLR signaling pathways in gut epithelial cells and immune cells, which help in maintaining intestinal health and improve gut-liver axis communication (153). Clinical studies have demonstrated the efficacy of phytobiotics in modulating the gut microbiome and enhancing liver detoxification, providing specific examples of their beneficial effects. For example, a clinical study evaluated the effects of curcumin on patients with NAFLD. The study found that curcumin supplementation significantly reduced liver fat content, serum AST, and ALT levels (154). The improvement was attributed to curcumin’s ability to modulate gut microbiota composition and enhance SCFA production, which positively influenced gut barrier integrity and reduced systemic inflammation. Another study investigated the impact of resveratrol, a polyphenol found in grapes, on patients with NAFLD. Resveratrol supplementation led to a significant reduction in liver enzymes, hepatic steatosis, and inflammatory markers (155). Mechanistically, resveratrol was shown to activate the Nrf2 pathway, enhancing the expression of antioxidant and detoxification enzymes in the liver (156, 157). Additionally, resveratrol modulated gut microbiota composition, increasing the abundance of beneficial bacteria such as Bifidobacterium and Lactobacillus (158). Another compound called lycopene which is derived from carotenoids, is linked to its ability to modulate several signaling pathways. Lycopene has been found to upregulate Nrf2 pathway, leading to increased expression of antioxidant enzymes such as SOD, catalase, and GPx (159–161). This upregulation helps mitigate oxidative stress.

However, *in vivo* efficacy is severely limited by poor bioavailability of some Phytobiotics and Herbal (162, 163). For example, Curcumin oral bioavailability in humans is less than 1% due to rapid glucuronidation and sulfation in the intestine and liver (164). Peak plasma concentrations after standard oral doses are in the low nanomolar range (typically 5–50 nM), far below the micromolar concentrations (1–50 μM) used in most *in vitro* studies (165, 166). This discrepancy fundamentally limits the translational relevance of many *in vitro* findings. Therefore, several approaches have been developed to overcome these limitations. Co-administration with piperine (black pepper extract) increases curcumin bioavailability by up to 2000% in humans by inhibiting glucuronidation (167). Lipid-based formulations, including nanoparticles, liposomes, and self-emulsifying drug delivery systems, improve carotenoid and polyphenol absorption (168). Co-administration with oils or fat-containing meals enhances lycopene absorption (161). However, even with these strategies, plasma concentrations of free parent compounds rarely reach those used in typical in vitro experiments (56).

Given the central role of the Nrf2-Keap1 pathway in hepatic detoxification and antioxidant defense, a growing number of natural compounds have been investigated for their ability to positively modulate Nrf2 expression and activity. (Table 5) provides a structured summary of an examples of these compounds, categorized by chemical class, with their proposed mechanisms of Nrf2 modulation.

**Table 5:** Natural compounds that modulate Nrf2 signaling.

Natural products	Proposed mechanism of Nrf2 modulation	References
Curcumin (Polyphenol)	Dissociates Nrf2 from Keap1 by modifying Keap1 cysteine residues; promotes Nrf2 nuclear translocation	(225)
Lycopene (Carotenoid)	Activates Nrf2 via ERK1/2 and PI3K/Akt signaling; upregulates Nrf2 target genes (HO-1, NQO1, GST)	(159)
Quercetin (Flavonoid)	Activates Nrf2 via MAPK (ERK1/2, p38) and PKC pathways; increases Nrf2 nuclear accumulation	(226)
Sulforaphane (Isothiocyanate)	Potent Keap1 alkylator; directly modifies Keap1 cysteine residues; most potent natural Nrf2 activator	(227)
*Lactobacillus plantarum* (Probiotic)	The *L. plantarum*-applesauce augmented NF-E2-related factor 2 (Nrf2) in the ischemic myocardium and induced Nrf2-regulated antioxidant enzymes heme oxygenase-1 (HO-1), NADPH dehydrogenase quinone 1 (NQO-1), and thioredoxin reductase (TRXR-1).	(111)

## 7 Gut–liver–hypothalamus axis and emerging mitigation strategies

### 7.1 A systems-level perspective on mycotoxin toxicity along the gut–liver–hypothalamus axis

This review highlights the complex and systemic nature of mycotoxin toxicity, moving beyond a single-organ perspective toward an integrated “gut–liver–hypothalamus axis.” Current evidence indicates that mycotoxins such as aflatoxins, ochratoxins, and deoxynivalenol (DON) not only cause localized damage but also initiate a pathological cascade that begins with intestinal dysbiosis and culminates in systemic hormonal and neurological disruption.

Began from mycotoxin-induced intestinal dysbiosis and disruption of tight junction proteins (e.g., occludin, claudins), leading to increased intestinal permeability (“leaky gut”). Then the translocation of mycotoxins and microbial products (e.g., LPS) to the liver via the portal vein triggers hepatic inflammation, oxidative stress, and Nrf2-mediated detoxification responses. In contrast, the subsequent link from liver dysfunction to hypothalamic disruption is less firmly established. While several studies report altered hypothalamic neuropeptide expression (e.g., POMC, NPY, GnRH) and behavioral changes following mycotoxin exposure. The direct evidence of mycotoxin or its metabolites crossing the blood–brain barrier (BBB) to reach the hypothalamic tissue (169). Most existing data are correlational, often based on indirect markers such as systemic cytokines or peripheral hormone levels. Direct quantification of mycotoxin levels in hypothalamic tissue following oral exposure, coupled with BBB permeability studies, is scarce (170). Therefore, while the gut–liver–hypothalamus axis represents a valuable conceptual framework, it should be viewed as a plausible pathway requiring further mechanistic validation, rather than a conclusively proven causal cascade.

### 7.2 Intestinal barrier disruption and initiation of the toxic cascade

Available studies indicate that the gut serves as the first line of defense against toxins. Mycotoxins disrupt this barrier primarily by reducing the expression of tight junction (TJ) proteins, including claudins and occludin (171), often mediated by increased levels of pro-inflammatory cytokines such as TNF-*α* and IL-6 (172). This “leaky gut” phenomenon represents a critical turning point, as it facilitates the translocation of both mycotoxins and microbial products such as lipopolysaccharides (LPS) into the portal circulation (173). Upon reaching the liver, these toxic compounds can overwhelm phase I and phase II detoxification pathways (174).

### 7.3 Hepatic oxidative stress and the protective role of Nrf2 signaling

A key aspect highlighted in this review is the central role of the Nrf2-Keap1-ARE signaling pathway as a major regulator of the hepatic antioxidant response. Natural products, particularly probiotics and antioxidants, appear to exert protective effects by stabilizing Nrf2 (175), thereby enhancing the expression of endogenous antioxidant enzymes such as superoxide dismutase (SOD), catalase, and glutathione peroxidase (176). Targeting the Nrf2 pathway may therefore represent a viable therapeutic strategy to mitigate mycotoxin-induced oxidative stress and prevent the progression to liver fibrosis or cirrhosis.

### 7.4 Neuroendocrine dysregulation and hypothalamic involvement

An additional focus is the emerging concept of the “gut–liver–hypothalamus axis.” Evidence suggests that liver dysfunction, combined with systemic inflammation, may permit toxic substances to cross the blood–brain barrier and affect hypothalamic function. Disruption of this central regulatory system, including alterations in TRH, CRH, and GnRH secretion, provides a mechanistic explanation for the broader physiological consequences of mycotoxin exposure (177), such as growth retardation, reproductive disorders, and appetite dysregulation (178–180).

### 7.5 Natural compounds and microbiota-targeted mitigation strategies

The growing interest in natural products—including probiotics (e.g., Lactobacillus and Bifidobacterium), prebiotics, and plant-derived compounds such as curcumin and lycopene—represents a promising direction in food and feed safety. Probiotics, in particular, offer multifaceted benefits by restoring microbial balance, producing SCFAs that enhance intestinal barrier integrity (181), and modulating immune responses via Toll-like receptor (TLR) signaling (182). These findings support the potential application of precision nutrition strategies aimed at reducing mycotoxin bioavailability before systemic absorption.

## 8 Challenges, limitations, and future research directions

A major limitation of the current literature and one that must be addressed in future researches is the inappropriate extrapolation of data across species when studying the gut-liver axis. Given these substantial interspecies differences, researchers should avoid making direct quantitative comparisons across species, specify the species, strain, age, sex, and dose regimen clearly, avoid generalizing findings from one species to another without direct experimental validation.

Despite these promising developments, several challenges remain. The efficacy of natural products is often influenced by strain specificity, dosage, and the bioavailability of active compounds. Although the Nrf2 pathway is a promising therapeutic target, the long-term consequences of its chronic activation require further investigation. Future research should prioritize the development of synergistic synbiotic formulations, combining probiotics and prebiotics with plant-derived compounds, tailored to specific mycotoxin exposures. For example, recent findings indicate that combined supplementation with lycopene and L-carnitine enhances lycopene bioavailability and upregulates genes associated with nutrient absorption and tight junction integrity (161). Additionally, further exploration of the bidirectional communication within the axis particularly the impact of hypothalamic stress signaling on gut microbiota remains essential. Well-designed clinical and field studies are also needed to establish standardized dosages and validate efficacy in both human and animal systems.

## 9 Conclusion

This review provides a comprehensive overview of the mechanisms underlying liver detoxification and its central role within the gut–liver–hypothalamus axis. The interconnected molecular pathways and signaling networks involved in this axis highlight its importance in maintaining systemic homeostasis under conditions of environmental toxin exposure. Disruption of gut microbiota balance represents a critical early event in mycotoxin toxicity, leading to compromised intestinal integrity, increased permeability, and subsequent translocation of toxins to the liver. This cascade adversely affects hepatic function and contributes to dysregulation of hypothalamic hormone secretion. Maintaining gut barrier integrity and microbial homeostasis is therefore essential for protecting overall physiological function. Natural products have emerged as promising therapeutic agents for mitigating mycotoxin-induced damage. Their ability to enhance gut integrity, support liver detoxification processes, and modulate systemic responses underscores their potential within the context of the gut–liver–hypothalamus axis. Nevertheless, continued research is required to further elucidate their mechanisms of action, optimize their efficacy, and explore their broader health benefits. Advancing this field may contribute to improved disease prevention strategies and overall health outcomes in both humans and animals.
